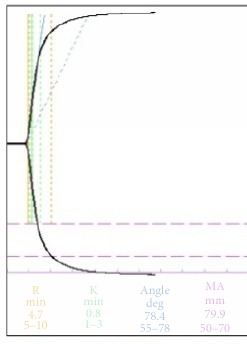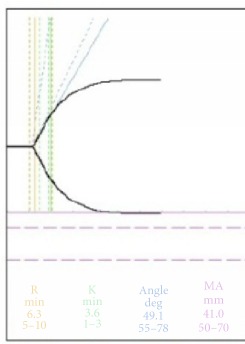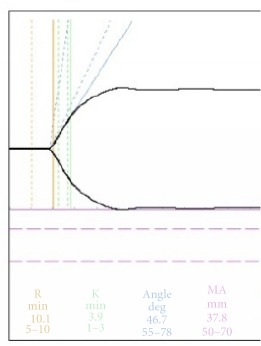# Use of Thromboelastogram in Venovenous Extracorporeal Membrane Oxygenation for a Patient with Pulmonary Hemorrhage due to Microscopic Polyangiitis

**DOI:** 10.1155/2019/7241264

**Published:** 2019-03-25

**Authors:** Chak-Kwan Tong, Jun Jin, Qian Du

**Affiliations:** ^1^Department of Anaesthesiology, University of Hong Kong, China; ^2^Intensive Care Unit, University of Hong Kong, Shenzhen Hospital, China

## Abstract

Systemic heparinisation is required for extracorporeal membrane oxygenation therapy, to prevent clotting of circuit and formation of thrombus in patient. Activated clotting time (ACT) or activated partial thromboplastin time (aPTT) has been the mainstay of monitoring of heparin dose. Thromboelastogram (TEG) is increasingly being used again in recent years with the advancement in technology. Its clinical usefulness in the monitoring of anticoagulation of ECMO therapy is demonstrated in the case presented. Our patient suffered from severe hemoptysis due to active microscopic polyangiitis and respiratory failure. Heparin infusion was given at the initiation of ECMO support without further aggravation of hemoptysis. Dose of heparin was adjusted successfully with the integration of the clotting profile and TEG results.

## 1. Introduction

Venovenous extracorporeal membrane oxygenation (VV-ECMO) support has been increasingly used in respiratory failure due to various causes. It is an organ support modality that attracts much attention since the 2009 Human Swine Influenza epidemic. One of the major complications of this invasive treatment is hemorrhage, related to the use of systemic anticoagulation, patient factor, and circuit factor. We report a patient who suffered from pulmonary hemorrhage and refractory respiratory failure due to microscopic polyangiitis and was given VV-ECMO support. Systemic heparin was used as anticoagulant, guided with activated plasma prothrombin time (aPTT), and thromboelastogram.

## 2. Case Description

A 60-year-old lady with satisfactory premorbid state, presented with dry cough for recent few months. She was admitted to the hospital in mid-Dec 2018 because of abdominal pain, joint pain, shortness of breath, and fever. She was noted to have renal impairment (serum Creatinine 538*μ*mol/L) on presentation. She had normocytic normochromic anemia, hemoglobin level 7.1g/dL, and elevated erythrocyte sediment ratio (ESR) of 130 mm/h. Radiological studies revealed bilateral lung infiltrates and normal-looking kidneys. There was mild proteinuria. Autoantibody testing showed positive antineutrophil cytoplasmic antibody (ANCA) and markedly elevated anti-PR3 antibody titer. Anti-GBM antibody was negative. Microbiological studies did not yield any positive bacterial culture, although her urine Streptococcal antigen was positive. She developed hemoptysis and respiratory failure 2 days after her hospitalization was and transferred to ICU for further care.

She was assessed by the Rheumatologist and suspected to have microscopic polyangiitis. She was advised to receive plasmapheresis, pulse steroid, iv IG, and cyclophosphamide. She was also covered with broad spectrum antimicrobial regimen.

Echocardiogram showed normal ventricular function and no valvular lesions. Bronchoscopy was performed in ICU showing diffuse blood-stained fluid from both sides of the airway. There was no endobronchial lesion. Due to the pulmonary hemorrhage, she had persistent desaturation (<80%) after ICU admission, despite escalation in mechanical ventilator support. The Murray's score was 3.7. Venovenous extracorporeal membrane oxygenation support was decided, to bridge for the effect of the immunosuppressive therapy. Her oxygenation improved right after the ECMO support was initiated. Blood flow rate was 3.5L/min.

As the Thromboelastogram (TEG) upon ICU admission showed hypercoagulable state, tight heparin was started upon initiation of ECMO with a target of 45-50s. TEG and aPTT were repeated for monitoring of the clotting status. The TEG and corresponding aPTT were depicted in Table 1. There was increase in hemoptysis on day 3 of ECMO support, and aPTT was <60s which was within the therapeutic range for anticoagulation. However, the TEG suggested worsening of coagulopathy, as compared with TEG of previous day. Heparin dose was reduced and hemoptysis improved.

Plasmapheresis and continuous venovenous hemofiltration were continued during ECMO support. Hemoptysis subsided on day 4 of ECMO support. Lung infiltrate improved from day 4 and urine output improved on day 5. ECMO support was weaned off on day 7. Patient was extubated 3 days after ECMO decannulation.

## 3. Discussion

Use of VV-ECMO as a bridge therapy for pulmonary hemorrhage due to vasculitis has been reported in case reports [1–3]. This case report serves to demonstrate another successful case. However, use of anticoagulation was not described in details in previous case reports. In our hospital, Thromboelastogram is available while the newer ROTEM is not available. Since this patient has pulmonary hemorrhage, better monitoring of the clotting status would be necessary and TEG [4] was added to the conventional clotting tests. Viscoelastic monitoring has been used in complicated conditions, like cardiac surgery [5] and trauma coagulopathy. Algorithm has been developed according to the results of the viscoelastic tests, especially ROTEM. Transfusion of various blood products becomes precise, which also results in reduction of transfusion in blood product and hence transfusion associated problems [6].

There is no standard recommendation on the use of anticoagulant when there is active bleeding during ECMO support [7]. Although this patient had active pulmonary hemorrhage, the conventional clotting tests were deranged and her TEG showed a hypercoagulable state. This was the reason for starting heparin infusion at initiation of ECMO support. Without the TEG result, other caregivers may choose not to start heparin infusion in the first 24 hours. This may pose potential threat of clotting of the circuit.

Interpretation of the various values of the TEG signifies the status of various components of the clotting pathway. Reaction time (R time) refers to the time it takes a new clot to begin forming. A prolonged R time indicates the abnormal overall functionality of clotting factors leading up to the conversion of prothrombin to thrombin. Transfusion of plasma or prothrombin complex concentrate can correct the abnormality, if indicated. Maximum amplitude (MA) reflects the strength of the clot, which depends on fibrin and platelet. Durability of blood clot is reflected by the lysis at 30 mins (LY30) or lysis at 60 mins (LY 60). Hyperfibrinolysis can be managed by using tranexamic acid.

On day 3 of ECMO support for our patient, there was an increase in hemoptysis after an increase in the dose of heparin infusion. Her aPTT was <60s while the TEG showed a significant worsening of the coagulopathy. However, as TEG-heparinase was not available in the hospital and the fibrinogen level and platelet count were 77x10^9^/L, it is decided that heparin dose should be reduced. Subsequent TEG improved and pulmonary hemorrhage subsided. Without TEG result, the increase in pulmonary hemorrhage maybe attributed to failure of the immunosuppressive therapy.

## 4. Conclusion

With the increasing use of VV-ECMO support, it is important that the risk of the treatment be minimized. For the bleeding complication, addition of clotting study to conventional tests could potentially help in the adjustment of anticoagulation and use of blood products during ECMO treatment.

## Figures and Tables

**Table 1:** Clotting profile.

	Before ECMO	Heparin infusion during ECMO Day 1	Hemoptysis recurred during ECMO Day 3

aPTT (s)	44	47.9	55

Platelet (x10^9^/L)	229	119	77

TEG